# The social context of wild leafy vegetables uses in Shiri, Daghestan

**DOI:** 10.1186/s13002-015-0047-x

**Published:** 2015-08-11

**Authors:** Iwona Kaliszewska, Iwona Kołodziejska-Degórska

**Affiliations:** Institute of Ethnology and Cultural Anthropology, University of Warsaw, Ul. Żurawia 4, 00-503 Warsaw, Poland; Faculty of Biology, University of Warsaw Botanic Garden, Al. Ujazdowskie 4, 00-478 Warsaw, Poland; Faculty of “Artes Liberales”, University of Warsaw, Ul. Nowy Świat 69, 00-046 Warsaw, Poland

## Abstract

**Background:**

Shiri is a small mountainous village in the Republic of Daghestan, in the North Caucasus. Daghestan is Russia’s southernmost and most ethnically and linguistically diverse republic, a considerable part of which belongs to the Caucasus Biodiversity Hotspot.

Various species of wild leafy vegetables are collected in Shiri and there are still many social and cultural practices connected with plant collection in the village. Yet due to migration processes, local knowledge about wild greens and their uses is being slowly forgotten or not passed on. The Shiri language is highly endangered and so are the local plant terminologies and classifications. The unstable political situation hinders local and international research, therefore we find it highly important to explore both what wild leafy vegetables are collected in this mountainous part of Daghestan and how the relation between plants and people is shaped in this linguistically and culturally diverse context. We answer the following questions: what wild leafy vegetables are collected in Shiri? Why are they important to the local people? What is the social aspect of wild leafy vegetable uses?

**Methods:**

The methods applied were as follows: forest walks and semi-structured interviews with adult inhabitants of Shiri village, participant and non-participant observation. During the walks herbarium specimens were collected, and visual recording of plant collecting process was conducted. This article is based on fieldwork done in Shiri, Daghestan, between 2012 and 2014, over the course of 3 field trips that took place in 3 seasons.

**Results:**

We collected and identified twenty-two local (24 botanical) species of wild leafy vegetables. Fourteen local species were used as snacks, eight for cooked dishes and three of them were also dried in order to be transported to kin living in the lowlands. It is significant that 70 % of taxa collected in Shiri are used as snacks. While snacks were collected by both sexes, greens for cooking and drying were part of the women’s knowledge.

The analysis of people-plant relations showed that care practices constitute an important part of these relations. Through the giving of wild greens, Shiri people express care for co-villagers and migrants and show their respect for elders. In the narratives about wild greens, their nutritional and taste value as well as perceived exceptionality were emphasized.

**Conclusions:**

1) Wild leafy vegetables are a significant element of everyday social life in Shiri in regard to mutual care, respect for elders and local identity. 2) Gender has a greater influence on practical skills than on declarative plant knowledge. 3) Names of plants are publicly discussed with elders and are not always fixed. 4) The moral value ascribed to giving in the local culture is expressed through wild leafy vegetables. 5) Care expressed through sending wild leafy vegetables helps to sustain social ties between migrants and Shiri inhabitants. 6) Identity, health and naturalness discourses are adding value to the local knowledge about wild leafy vegetables.

**Electronic supplementary material:**

The online version of this article (doi:10.1186/s13002-015-0047-x) contains supplementary material, which is available to authorized users.

## Introduction

Shiri is a small mountainous village in the North Caucasus in the Republic of Daghestan, the southernmost and the most multiethnic republic in the Russian Federation [see map Additional file 1]. The research area belongs to the Caucasus Biodiversity Hotspot (WWF and Conservation International). There are over 6500 species of vascular plants, around 25 % of which are local endemics [1]. Simultaneously, it is a place of high cultural and linguistic diversity.

In the Daghestani highlands there are many social and cultural practices associated with plant collection. Therefore, we find it important to understand the relationships between people and plants. Various species of wild leafy vegetables are collected by local inhabitants in the area surrounding Shiri. While knowledge about those plants is still prolific, it is slowly being forgotten or not passed on, due to migration processes and low interest among the younger generations. To understand the cultural role of wild leafy vegetable uses in a given settlement, it is important to learn not only about the species and their uses, but also about the social aspect of practices linked to their collection.

During our research, we tried to answer the following questions: what species of wild leafy vegetables are collected and why? What are they used for? Why are leafy vegetables important to the local people? What makes them an object of desire for migrants?

In this article we will describe what species of wild plants are collected as leafy vegetables in Shiri and what makes them important to the local people. We will discuss both the local practices and the narratives. We will show the social context of leafy vegetable collection. We see such context of plant collection as critical component of people-plant relationships that are often omitted in ethnobotanical analysis (cf. Howard on gender relations [2]) and are not widely discussed in the literature. Therefore, we find it important to undertake this discussion.

Daghestan and its social and botanical diversity have not received much academic attention in recent years. It is one of the last places in the Caucasus where people live on the slopes of the mountains, sometimes in very remote villages where wild leafy vegetables constitute an important element of their diet. While this is likely to change in the coming years due to migration, we found it important to take a closer look at wild leafy vegetables, their uses and social significance. Besides, as for example Rivera and colleagues point out studying wild vegetables may add to the knowledge of food sciences and toxicology [3].

Daghestan with its high contemporary biocultural diversity and early agriculture development is a very interesting research spot for ethnobiologists but, according to our knowledge ethnobotanical research has not been done in the region. According to the survey on medical ethnobotany in Europe (including the Caucasus) done by Quave, Pardo-de-Santayana and Pieroni, most of the research in Europe is conducted in Mediterranean regions including the Balkans, while no work was cited from the Caucasus [4]. The reason for this may be that the Caucasus is sometimes regarded as a part of Europe and sometimes as a part of Asia, Central Asia in particular. There is no published contemporary ethnobotanical research based on fieldwork from Daghestan. Hardly any research has been done in other parts of the Caucasus. There is a recent ethnobotanical survey from Georgia done by an international team of researchers, and the work on the subject is going to be continued [5]. In their analysis of Mediterranean gathered food plants, Rivera and colleagues present a comparison with GFP in the Caucasian diet and they admit that there is not enough data on the subject. Their main source is the work “Rastitel’nye Bogatstva Kavkaza,” by Grossheim, published in 1952, which enumerates 500 gathered food plants for the whole Caucasus [3]. There is an interesting work in Russian on wild food plants in the traditional cuisine of the Kabardinians. The book was edited in 2003 by Shkhagapsoiev, Shorova and Kozhkov, and sums up the work on the subject from the 1970s–2002 [6].

## Background

Daghestan is the most unstable republic of the Russian Federation, torn by internal political and social conflicts. Conflicts between power structures and the so-called Islamic underground, corruption, crime and overpopulation are but a few problems the republic faces (more [7]). Most Dagestanis are Sunni Muslims and, in recent years, many have become more observant with regard to nutrition.

The Caucasus, and Daghestan in particular, is the place with the greatest linguistic variation in Europe, so it is not only a botanical but also a linguistic ‘residual zone’ – a place whose geographical characteristics are crucial for the survival of many language families in a small area [8]. Shiri village is located in the mountains in the Dakhadaevsky region of Daghestan, near Kubachi, a bigger settlement famous for its production of silver jewelery. People in Shiri speak a distinct language which belongs to the Kubachi subgroup of the Dargi languages [9] and has not been documented until recently.^1^

The village of Shiri is situated on the northern slope of the mountain at the altitude of around 1500 m above sea level [see Additional file 2]. There are only eleven households in the village, seven of which are inhabited by Shiri people, and two by so-called newcomers. Some of them have been living in the village for over ten years, yet arrival from a different village (Tselibki) results in their “guest” status. There are also two mixed households. Over the three years that our research was conducted, some households changed dwellers, and two Shiri inhabitants died. Two households (with one elder each) were inhabited only from spring to autumn, while winter was spent in the lowlands, where access to medical care and shops is easier. In the event of snowfall or excessive rain, the road from Shiri to Kubachi is impassable. In other seasons it is accessible only by a four-wheel-drive vehicle. There is a mosque in the village and a small elementary school from first to fourth grade, with five students and two teachers. Older children are send to relatives in other settlements with better access to educational facilities. Most inhabitants of Shiri live off old age or disability pensions, cattle-breeding, cheese making or beekeeping (2 persons).

In Soviet times, people tried to grow vegetables in small gardens near their homes, yet nowadays only three of these plots are maintained. “Vegetables do not grow here as well as they do in the lowlands. And the Colorado potato beetle [(*Leptinotarsa decemlineata*)] eats them up,” one woman explained “I only keep potatoes and carrots for the children because it is ecological, clean of fertilizers”. Due to the economic growth in Russia in the 2000s, most inhabitants of Shiri can afford to buy potatoes, onions, carrots and occasionally cucumbers and tomatoes in nearby Kubachi. Watermelons and melons are brought to Shiri by minivan once in the season.

The number of people in Shiri is constantly decreasing from year to year. In the late 1950s, after returning from Chechnya, where the inhabitants of Shiri were forcibly resettled in 1944 by Soviet authorities (after the deportation of Chechens to Central Asia), many, seeing their houses destroyed, were both forced and encouraged by the state to move to the collective farms (*kolkhoz*) in Chinar (Derbentsky District) and Druzhba (Kayakentsky District), or to cities in the republic of Daghestan (Makhachkala, Derbent, Izberbash) and elsewhere.

Most of current inhabitants of Shiri were born either in Chechnya or in Daghestan shortly before or after the deportation to the Chechen village of Mayurtup. Some of them have learned about edible greens only in the late 1950s after coming back from Chechnya. They have some memory of collecting wild fruits in the forests of Chechnya but none of them remembered collecting the leafy greens. The parents of our interlocutors, who experienced famine during the 30s and 40s, might have collected edible greens as famine foods, yet the contemporary inhabitants of Shiri do not consider them as such and do not remember stories about collecting wild greens because of the famine.

The collapse of infrastructure following the fall of the Soviet Union has left many of them unemployed, resulting in seasonal migration to Moscow and other bigger cities outside of Daghestan.

Nowadays elders from Shiri are encouraged by their children to join them in the lowlands, while the younger generation looks for permanent or seasonal work outside the republic. Similar threats to biocultural diversity were found in mountainous regions of Georgia (Caucasus) in the survey conducted by Bussmann and colleagues in Georgia [5] after [11].

In the lowlands, Shiri speakers usually switch to Russian to communicate with neighbors of different ethnicity, while their knowledge of the standard Dargi language, based on Akusha-Dargwa, is usually limited to what they have learned at school [10].

Once a year, around June 21, many migrants of Shiri origin visit Shiri to perform *mavlid* (recitations of specially written poems to commemorate the Prophet Mohammad’s birthday, also practiced on special occasions in Daghestan, like births, weddings, funerals, etc.) It is performed at the shrine (*ziyarat*) of sheikh Hasan, and visitors donate some money for the community. Wealthy migrants sponsor renovations of the *ziyarat*, mosque or other significant places in Shiri. In Daghestan the possession of goods or money obligates one to share with kin and co-villagers.

Knowledge about plants, including wild leafy vegetables, is most common among women, cf. [12]. It is mostly girls who are socialized to traditional women’s roles (cooking, housekeeping, child-rearing). They learn about wild leafy-vegetables used in the kitchen from older women in the family. Yet, knowledge about wild leafy vegetables eaten on the spot (snacks) is also shared among boys, who are more often than girls send up to herd cattle, which is traditionally considered a man’s job.

Traditional knowledge about wild leafy vegetables is only partially passed on to members of the younger generation, most of whom visit Shiri only in summer. Due to migration to multiethnic settlements, the Shiri language is gradually dying out, along with the people’s knowledge of plants. A majority of the wild leafy vegetables available in Shiri are not available in the dry lowlands, so uses and names are being forgotten.

### Methodology

This article is based on fieldwork conducted in Shiri, Daghestan, between 2012 and 2014. There were three field trips in May, June, July and August, altogether eight weeks of fieldwork. Wild leafy vegetables are most commonly collected in this period. The research period was chosen based on the first author’s previous numerous visits to other regions of Daghestan (altogether 1.7 years in the field). Interviews and trips were video-recorded in order to grasp both what is collected and how it is done. The authors’ experience of fieldwork in Daghestan, in 2004–2014, was useful for a broader cultural context [7, 13].

Our methodology was based on participant and non-participant observation, forest walks with 5 key-informants (all of them female, aged approximately from 40 to 90) and several semistructured interviews with each of 10 adult female and 5 male inhabitants of Shiri village, all of them non-specialists in plant knowledge. The age range of informants was from the mid 30s to 90s, however the older inhabitants’ knowledge was visibly deeper and they were more eager to share it.

There was at least one person interviewed from each household in the village. Each interview was voice-recorded, and all of the forest walks were video-recorded. Food preparation, plant sorting at home and some of the interviews were also video-recorded (see Video 1 on *ħuˤlkni* with leafy vegetable preparation). During the interviews and walks, we asked about current plant use, but we did not focus on historical data or memories on former plant use. Before each interview verbal informed consent was obtained.

The main language of research was Russian and Shiri. All but two of the inhabitants of Shiri spoke Russian well enough to prefer it as a language of communication without the need to speak via interpreter. Russian is the *lingua-franca* in Daghestan and it is the most common language of communication with people outside of the village. Shiri is spoken only in the village (and in the lowlands where Shiri people migrated) while mutually comprehensible languages are only found in some nearby settlements. Translation was needed during one forest walk, while interviews were recorded both in Russian and in Shiri, depending on the speaker’s will and ability to communicate comprehensibly in Russian. Shiri language has never been documented before so interviews in Shiri were part of the linguistic documentation of the project.

Voucher specimens were collected during the forest walks and were deposited in the herbarium of the University of Warsaw Botanic Garden. Plant and author names were verified according to The International Plant Name Index (http://www.ipni.org/) and The Plant List (http://www.theplantlist.org). Local Shiri names were transcribed according to the standard transcription for Caucasian languages. Research was done in accordance with the ISE code of ethics, where appropriate.

## Results and discussion

### What is collected?

Wild leafy vegetables are used in Shiri in three ways: for cooking, as snacks and for drying (Table 1). There are 22 folk species (24 botanical taxa) of leafy vegetables and edible flowers collected in the village. Sixteen of them (72 %) are used as snacks: eaten raw, eight (36 %) are cooked, three of which are also dried to be send to the lowlands. Four of the folk plant taxa have multiple culinary uses, which is why percents do not add up to 100 %. Most of the species were enumerated by a majority of the interlocutors. Even if only one person in the village actually collected a certain taxon, many others, if not all the others, knew about it because leafy vegetables brought from the field were often spoken about at the central meeting point in the village (discussed further). Knowledge of medical plants was more individualized, but this case exceeds the scope of this article.

**Table 1:** Wild leafy vegetables collected in Shiri, Daghestan. The most popular leafy vegetables collected in Shiri during field walks are presented in the table. Local names shown in the table are the most widespread ones

Local name	Latin name	Plant family	Plant part	Usage	Herbarium number
sːisːupi	*Allium victorialis* L.	Amaryllidaceae	leaves	cooking, drying, snack	WABG 002645
birikːa	*Anthriscus cerefolium* (L.) Hoffm.	Apiaceae	leaf stalk	snack	WABG 002652
ʁˤupːisqʷa	*Anthriscus* sp.	Apiaceae	leaf stalk	snack	WABG 002662
haq’ul	*Arctium lappa* L.	Asteraceae	stem, root (young, first year)	snack	WABG 002663
duc’armura	*Bunias orientalis* L.	Brassicaceae	flowers	snack	WABG 002646
qːaʁa	*Cephalaria transsylvanica* (L.) Schrader ex Roem. et Schult.	Dipsacaceae	leaves	cooking	WABG 002659
ʡaˤʁʷamura	*Cerastium davuricum* Fisch. ex Spreng.	Caryophyllaceae	leaves	cooking, drying	WABG 002660
biričːa	*Chaerophyllum aureum* L.	Apiaceae	leaf stalk	snack	WABG 002654
bah gʷa̰gʷa̰	*Crocus reticulatus* Steven ex Adam	Iridaceae	flowers	snack	no herbarium specimen
pilalaw:ti	*Fritillaria collina* Adam	Liliaceae	flowers	snack	no herbarium specimen
podsniezhnik (rus.) bah gʷa̰gʷa̰	*Galanthus* spp.	Amaryllidaceae	flowers bulbs	snack	no herbarium specimen
q’aramura	*Galega orientalis* Lam.	Leguminosae	leaves	snack	WABG 002650
ʁʷaža birikːa	*Heracleum apiifolium* Boiss.	Apiaceae	leaf stalk	snack	WABG 002651
qːaˤnala čutni	*Malva* spp. (*Malva neglecta* Wallr. and *M. pusilla* Sm.)	Malvaceae	leaves, stem, unripe fruit, nectar	cooking, snack (unripe fruit and nectar sucking)	WABG 002653 WABG 002665
guržinakːʷi	*Oberna multifida* (Adams) Ikonn.	Caryophyllaceae	leaves	cooking, drying	WABG 002661
q’urtabːisqʷa	*Pimpinella* cf. *major* (L.) Huds.	Apiaceae	leaf stalk	snack	WABG 002656
daga̰la qʼar	*Plantago major* L.	Plantaginaceae	leaves	cooking	WABG 002655
žibžni	*Polygonum aviculare* agg. *sensu lato* L.	Polygonaceae	stem with leaves	cooking	WABG 002649
piervocviet (rus.) bah gʷa̰gʷa̰	*Primula veris* subsp. *macrocalyx* (Bunge) Lüdi	Primulaceae	flowers	snack	WABG 002658
q’ac’miža	*Rumex acetosa* L.	Polygonaceae	leaves	snack	WABG 002657
sirič’ič’ni	*Sedum spurium* M. Bieb.	Crassulaceae	leaves	snack	WABG 002664
mec	*Urtica urens* L. and *U. dioica* L.	Urticaceae	leaves	cooking	WABG 002647 WABG 002648

*Snacks* not only eaten on the spot, but also brought back from the trip to share with co-villagers met in the street or at the *godekan* (central meeting point in the village). Collected by both adults and children

*Cooking ħuˤlkni* / *chudu* (pie with filling), *kurze* (dumplings) and soup

*Drying* only to send to the lowlands for *ħuˤlkni* or *kurze* fillings

Giving the number of wild leafy vegetables used in one small village (11 households), we are able to state that this is a herbophilous community [14]. Each interlocutor knew and used at least 10 species of wild leafy vegetables. This is all the more significant as this study focused only on wild greens and flowers that were eaten. We did not analyze data on recreational teas or wild fruits eaten by the community members, though they would undoubtedly add to the overall number of wild plants gathered by the Shiri people. During the first part of our research, we focused on wild greens, as they played a great social role (research on fruits and medical plants in Shiri is planned for 2016 and 2017). Though in many communities in Europe wild fruits or plants gathered for recreational teas are regarded by people as most important, here the trend is the opposite. For example, in Ukraine, Romania, many parts of Russia, and Estonia recreational teas make up the considerable part of edible plants gathered [15, 16]. Research conducted in the Caucasus shows many wild fruits gathered in Armenia [17]. Based on preliminary results, we can state that the number of wild fruits gathered by Shiri inhabitants is much lower. Europe’s Mediterranean region is usually seen as the area where people have a preference for wild greens [14], but Pardo-de-Santayana and colleagues note that even in many communities on the Iberian Peninsula, “There is a clear preference for wild edible fruits that are consumed raw or used to make jams and liqueurs. By contrast, people in most of the study areas reject many available wild vegetables [18]”. In Shiri teas made of wild plants (not *Camelia sinensis*) are treated as food for poor members of the community. It is shameful to give such tea to a guest, while giving wild greens is a source of pride. The number of gathered wild leafy plants used is similar to those noted by Bussmann and colleagues in Georgia [5] and by Shkhagapsoiev among Kabardinians [6].

### Cooked leafy vegetables

The most popular mode of preparation is cooking. Fillings of *ħuˤlkni* (pie with filling, also referred to as *chudu*) and *kurze* (dumplings) are made predominantly out of *guržinakːˤi* (*Oberna multifida*), *ʡaˤʁˤamura* (*Cerastium davuricum*), *qːaˤnala čutni* (*Malva neglecta* and *Malva pusilla*), *qːaʁa* (*Cephalaria transsylvanica*) or young *mec* (*Urtica urens* and *Urtica dioica*). Soup may be prepared from *sːisːupi* (*Allium victorialis*). Knowledge of wild growing leafy vegetables used for cooking is evenly spread among women in the village. Knowledge sharing is encouraged by collective cooking cf. [19]. Often, when *ħuˤlkni/chudu* or *kurze* is being prepared*,* women visitors are supposed to help because preparation of this dish is time consuming. The knowledge of these eight plants is so obvious for the women in the village that, in their eyes, it is barely worth talking about. Local plant names are shared.

One of the most typical examples of usage is the preparation of *ħuˤlkni. Qːaʁa* (*Cephalaria transsylvanica*) is shredded with a knife. Cottage cheese, salt, butter and eggs are added to make a filling, which is formed in balls (ca. ten cm in diameter). A round piece of dough fifteen cm in diameter is formed, and a ball of filling is put in the middle. The edges of the dough are carefully folded over the filling. The pie (*ħuˤlkni*) is rolled out and then put in the oven and baked. A similar filling may be used for the dumplings known as *kurze.* Other plants may be prepared in the same way; for the list of plants for *ħuˤlkni* or *kurze* see Table 1.

An additional movie file shows the making of *ħuˤlkni/chudu* of *qːaʁa* (*Cephalaria transsylvanica*) in more detail [see Additional file 3].

The days around June 21, when *mavlid* is performed at the shrine of sheikh Hasan, are the proper time for the collection of *guržinakːˤi* (*Oberna multifida*) and *ʡaˤʁˤamura* (*Cerastium davuricum*). These plants are the two most important plants collected by most women in the village. They are considered the most tasty and unique to this location (*O. multifida* grows only in the Caucasus and Artvin and Rize Provinces in Turkey [20]). According to our knowledge, this is first report of this plant being consumed by people. It is closely related to *Silene vulgaris* and other *Silene* species eaten in many parts of Europe and Asia (for example, the Iberian Peninsula [18], Turkey (Central Anatolia) [19]), as well as to *Oberna wallichiana,* eaten in Georgia in a very similar way [5]. The women from the lowlands who visit the shrine use the opportunity to go for a walk (if they manage to escape from cooking and serving the men) and collect some plants to take with them to the lowlands, or they eat the leafy vegetables on the spot. Some women also collect “new” plants they learned about from books, TV or the Internet cf. [21].

### Snacks

Snacks are usually eaten on the spot by both adults and children while going to or from the pastures. The amount of greens eaten is very individual, depending on hunger and the duration of the walk. Sometimes, if in a hurry to the pastures, it is only a couple of *duc’armura* (*Bunias orientalis* L.) flowers that are picked without even stopping. More greens are usually eaten on the way back from the pastures. Most often two or three stalks of *ʁˤaža birikːa* (*Heracleum apiifolium* Boiss.), *birikːa* (*Anthriscus cerefolium* (L.) Hoffm.) or *biričːa* (*Chaerophyllum aureum* L.) are peeled off and eaten, as well as a couple of leaves of *sirič’ič’ni* (*Sedum spurium* M. Bieb.), which grows near the village on the way to the pastures. On longer walks, more stops are usually made to eat snacks. *Sːisːupi (Allium victorialis* L.) may be eaten along with food (usually bread and cheese), though we never recorded wild greens being eaten in the form of a sandwich or salad.

Snacks may also be brought back from the field trip to share with co-villagers, especially those who can’t walk long distances. They are shared with people met in the street or at the *godekan,* a central meeting point in the village [see Additional file 4]. If snacks are brought home, they are usually eaten right away, typically before the dish is prepared and brought to the table.

The snack-plants we decided to present in Table 1. are not only leafy vegetables. We included plants collected for the consumption of their flowers (four species). Additionally, information about snacking on unripe *Malva* spp. (both species *M. neglecta* and *M. pusilla*) fruit has been added, although these plants is shown in the table mainly as a source of leaves and stems for cooking.

Knowledge about snacks is familiar to both women and men, but it is less evenly spread among the interlocutors than knowledge about plants used for *ħuˤlkni/chudu* and *kurze.* Some of the interlocutors didn't keep cows, so they had fewer occasions to practice this knowledge. Male interlocutors were much more interested in talking about these plants than those used for cooked dishes. This is connected to the gender division of labor in the village (compare for example [22]).

Most of wild greens collected by Shiri people are eaten as snacks (72 % of plants reported in the research). The consumption of salads with wild leafy vegetables (characteristic of Mediterranean cuisine e.g., [23, 24]) does not occur in the mountainous locations of Daghestan. Moreover, people in Shiri rarely eat other raw vegetables such as tomatoes and cucumbers bought in the shop. They are mainly served to guests or brought by kin from the lowlands, and are not consumed on an everyday basis. Similarly, in 19th-century Estonia the consumption of salads was limited, although many species of wild green vegetables were eaten [25].

Snacks in European ethnobotanical studies are usually associated with children. Florivory (eating flowers) in particular is seen as a phenomenon that is typical of kids [24, 26, 27]. According to Kalle and Sõukand, in 19th century Estonia snacks were eaten by both children and adults, but are now mainly consumed by children [25]. All the snacks in this survey were eaten by both adults and children. As the analysis of adult, non-specialist, contemporary knowledge of wild leafy plants was the goal of this study, we do not have data on snacks that are specifically eaten by children. Data on snacks collected by children comes only from observation. Moreover, children usually visit the homes of their relatives in the village during the summer holidays. We did observe flower eating by both adults and children (*bahgˤa̰gˤa̰/piervocviet, Primula macrocalyx; duc’armura, Bunias orientalis; bahgˤa̰gˤa̰/podsniezhnik, Galanthus* spp.; *bah gˤa̰gˤa̰, Crocus reticulatus*; *pilalaw:ti, Fritillaria collina*). The first two species are mentioned by Bussmann et al. *Bunias orientalis* – edible flowers, *P. veris* subsp. *macrocalyx* (Bunge) Lüdi as medicinal herb [5]. *Bunias orientalis* is also mentioned as eaten in the region by Shkhagapsoiev et al. [6]. Kabardinians used to eat bulbs of the *Galanthus* species, but Shkhagapsoiev et al. do not mention its flowers being eaten [6]. Although nectar sucking is considered to be a typical snack for children [27], we did observe adults (and only adults) sucking nectar from *Malva* spp. (*Malva neglecta and M. pusilla*) flowers. Nevertheless we did not notice any other flowers being sucked. This may be due to the fact that our main focus was on adults. The high number of snack-plants may be seen as the result of living in close contact with the local environment [28]. It might seem interesting that the flowers of potentially toxic plants are eaten – *Galanthus* spp., *Fritillaria collina*, *Crocus reticulatus.*^2^ Eating *Crocus* species as was reported, for example, in Turkey (*Crocus ancyrensis*) in Central Anatolia [19]. Various *Fritillaria* species (usually bulbs) are eaten for example in America [30] and Asia, for example China (bulbs and flowers) [31]. *Galanthus* spp. eating seems more rare, and we did not find any English source citing the edibility of this plant, although in the region it was eaten in Kabardino-Balkaria [6]. The eating of flowers of these species was mentioned by interlocutors much less frequently than the use of these plants in cooked dishes. They were not considered a source of local pride and were not connected with Shiri identity, as opposed to leaf stalks of the *Apiaceae* species (snacks) and *Oberna multifida* and *Cerastium davuricum* used in *chudu*. No information on them being famine food was mentioned. Three plant species flowering in early spring and having conspicuous flowers are mentioned under the same local name – *bah gˤa̰gˤa̰*. They are: *Crocus reticulatus*, *Galanthus* sp. and *Primula macrocalyx.* For both *P. macrocalyx* and *Galanthus sp.,* people also use common Russian names. More research is needed to explain this phenomenon. Undoubtedly these plants are considered by the locals as three different taxa, unless they have a common name. What is similar for them is usage type and phenology.

Apiaceae is the plant family best represented in the snack-plant use category. Out of 16 local taxons of snack-plants five are leaf stalks of Apiaceae family members. Leaf stalks of various Apiaceae species are highly valued and are longed for by those who left the village. Obviously, snack-plants can not be dried and sent down to the lowlands. Comparison of our results with data from other parts of Europe showed that Apiaceae are consumed as snacks in various communities in Europe and the Caucasus. For example, Bussmann and colleagues noted two *Heracleum* species as being consumed raw in Georgia; they are different species than the one consumed in Shiri [5]. Nedelcheva in Bulgaria observed the use of *Chaerophyllum bulbosum* (different species than in our data), *Anthriscus* spp., *Рastinaca sativa* (not present in our research) and *Heracleum sibiricum* (different species than in Shiri) [32]. Also Kabardinians (who live in environmental conditions most similar those in Shiri) eat different *Heracleum* species as snacks (*H. roseum*) [6].

### Drying

Drying is only performed with the purpose of sending the wild leafy vegetables to the lowlands. The most commonly dried plants are: *ʡaˤʁˤamura* (*Cerastium davuricum*), *guržinakːʷi* (*Oberna multifida*) and *sːisːupi* (*Allium victorialis*). One of these plants being very important for Shiri people – *A. victorialis* is also collected in Georgia [9]. It is gathered in huge quantities (as in Shiri) and is used cooked and also pickled. Also other *Oberna* species – *Oberna wallichiana* (Klotzsch) Ikonn. is eaten in Georgia [5].

### Contexts of importance of leafy vegetables to the locals

We decided to divide plant use contexts into practices performed by Shiri inhabitants and narratives about the wild leafy vegetables present in the village. We will start by taking a closer look at the practices, and then we will proceed to the narratives.

### Local practices

#### Care for relatives in the lowlands

Looking closer at the local practices of wild leafy vegetable uses shows that the significance of wild leafy vegetables in the life of the local villagers is closely linked with the moral value ascribed to giving in the local culture. The plant-sharing practices in Shiri include elements of care for relatives in the lowlands. Therefore, leafy vegetables play a significant role in sustaining good ties between people living in the lowlands and in the highlands. Care is expressed by sending down big piles of fresh or dried leafy vegetables for kin and friends. Plants were even collected for strangers who asked for them (see case 1).

##### Case 1

Patimat was in a hurry in the morning. Her grandson took his father’s car when the latter was away on seasonal work, and caused a car accident. Although nobody died in the crash, a boy was injured and Patimat’s husband, as the elder of the lineage, was supposed to go to the lowlands and visit the injured and offer him some money as compensation for the irresponsible behavior of his young grandson. It takes up to 5–6 h to go to the lowlands, if one leaves early enough to catch the minibus from Kubachi (nearest city-like settlement). There is no public transport from the village of Shiri. It is accessible only by four wheel drive.

Although the car accident was the main reason for her husband’s trip, Patimat used the opportunity to send a big pile of wild leafy vegetables collected the day before for her daughters living in the lowlands, in Izberbash. She sat down during the nightand sorted the weeds from *ʡaˤʁˤamura* (*Cerastrium davuricum*) and *guržinakːˤi* (*Oberna multifida*) to make it “clean”, ready to use for *ħuˤlkni* with greens, the favorite filling of this dish of one of her daughters. She did not pass any other gifts with her husband except for money and the plants. While gift-giving is an important practice in Daghestan and one should not go empty handed when visiting relatives, in recent years material objects (typically kitchen utensils, clothes, adornments) are gradually replaced by money. Our informants claim that this does not apply to plants. Although certain plants (for example *ʡaˤʁˤamura* (*Cerastrium davuricum*) and *guržinakːˤi* (*Oberna multifida*)) are highly requested and cannot be found in the lowlands, they are nevertheless not sold by Shiri people. Füsun Ertuğ observes similar attitudes in Anatolia in Turkey, where local women also do not consider wild greens marketable [19]. Some wild leafy vegetables are vended at the bazaar in Makchakala, but they are viewed by Shiri people as unfresh and “suspicious” because their origin is not known. The above makes plant packages even more needed (in the eyes of Shiri people who claim that their kin “live in dry land where there is nothing”).

A week later Patimat learned that her neighbor was going the Druzhba village in two days. This prompted day-long walk for *sːisːupi* (*Allium victorialis*). She told her husband that we would be away for the whole day, so he had to prepare his dinner by himself. Patimat packed bread and cheese and we set off after breakfast (most of the walks take place during the day, never too early in the morning). Her 10-year-old grandson, who came for holidays, joined us during the trip – he wanted to climb up the mountain with his newly constructed bow. We walked up the hill continuously for around 2.5 h, without stopping to pick plants on the way, until we made it above the tree line almost at the top of the mountain overlooking the village. “It is only here that we come for *sːisːupi*” she said, “I've never seen it anywhere else”. Collecting wild leafy vegetables for *ħuˤlkni* / *chudu* usually takes half an hour or less. *Sːisːupi* collecting turned out to be a “serious” endeavor. Patimat instructed her grandson and me how to collect it and which plants were good and which were bad (those in bloom were considered too old). She sat down and collected the sːisːupi around her three times faster than we did. After around 2 h, the piles of the plant were the size of a cow and I was starting to worry how we would to carry all of it down the steep slope [see Additional file 5].

“Isn’t it enough for *chudu* / *ħuˤlkni*?”, I asked. “No, we need some more. I will send it down (to the lowlands) with Ibragim and he will give it to Mariam,” Patimat said. “Is it going to stay fresh?”, I inquired. “Of course, we will cut it in pieces in the evening and dry it in the sun the next day. Then it can be used for the whole year [In the highlands it is usually eaten immediately after collection].”

Patimat took the bread and cheese out. “It contains a lot of vitamins! Here, eat like me!” She took *sːisːupi* and ate it interchangeably with bread and cheese.

“Have you ever thought of selling *sːisːupi* (*Allium victorialis*) or other greens/plants in the lowlands?”, I asked later while chatting with Patimat. “No!” She felt offended or startled by my question. “No, it wouldn’t be appropriate. I would be ashamed of doing it. even if I was short of money. If somebody asks, I will go and collect plants for them. Even if I don’t know those people. Sometimes they come from the town and ask for *ʡaˤʁˤamura* (*Cerastium davuricum*) and *guržinakːˤi* (*Oberna multifida*), as it grows only here. Then I go and collect it.”

It turns out that plant giving still remains one of those shrinking areas of life not regulated by money. Under no condition are wild leafy vegetables sold, even to strangers. Patimat and other women in the village confirmed that they heard that there were now some “strange” people selling greens for *ħuˤlkni* / *chudu* at the bazaar in Makchakala (the capital of Daghestan). There is nobody like that in Druzhba or Chinar. Besides, she or her kin would never buy it because “you don’t know where it comes from” or “you have no idea how long it’s been lying there or if it is fresh.” Both the proofed origin and reliability of the one who collected the plants are important. The “alien” origin is perceived as a threat to both health and taste (cf. [33, 34]).

The way women spoke about it may indicate a strong emotional link to plant giving. Another issue is the identity context: − the plant’s exclusiveness (it grows only here, it’s the most tasty, it’s ours). Through giving they express their care (more on care and food in the post-socialist context: [35]) and they feel needed cf. [36].

While in many cases those who “stayed behind in the mountains” receive remittances from kin from the lowlands (though in half of the cases households in Shiri were profitable from cattle breeding), plant and food (milk, cheese, dried cow meat) giving remains one of the domains where the villagers can reciprocate or simply make a valuable gift.

Women took pride in undertaking long walks and providing wild greens for their families and kin in the lowlands, yet it seems there was more to it. Longer walks may also be seen as an occasion to leave the house or socialize with other women. Similar practices were recorded by Füsun Ertuğ in Central Anatolia, Turkey [19]).

#### Mutual care for villagers and respect for elders

While coming back from the walk late in the afternoon, Patimat greeted Hadjilay, the eldest man in the village, who was sitting near the House of Culture, Soviet style community center. “What are you carrying on your back?”, he asked. “*Sːisːupi,”* Patimat answered. “I will give you some,” she said. “No, I don’t want that. I want *ħuˤlkni* with *sːisːupi,*” Hadjilay answered. Whether she planned it or not, apart from preparing *sːisːupi* to be taken to the lowlands, Patimat made *ħuˤlkni* with *sːisːupi* late in the evening*.* She also sent her granddaughter to give a couple of *ħuˤlkni* to Hadjilay (far more than he would be able to eat himself). He had been living alone since his wife died. He was occasionally visited by his kin, and women in the village took care of him, bringing him some food everyday.

Wild leafy vegetables play a significant role in the social relations in the village. Snacks brought from the fields are shared with people met on the way or with those sitting at the *godekan* (central meeting point in the village). If special food is made, like *ħuˤlkni/chudu* with *sːisːupi* (collected far away from the village), those met on the way from the trip are either invited to eat or the food may be brought for them as *sadaqah* (voluntary gift in Islam). Leafy vegetables used for *ħuˤlkni* are also collected for those villagers who cannot collect them by themselves.

##### Case 2

There is a shift system for taking care of cows in Shiri: the number of cows equals the number of days their owner is required to take them to pasture. After dawn it is Malaykat’s turn to take care of the cows of the whole village. Most of the cows belong to her household. We walk along the edge of the village, Malaykat is busy keeping the cows together, occasionally yelling or patting them. She then separates the heifers from the older cows, which continue walking down and then up the hill. We cross a little stream in the valley. Malaykat walks in and struggles with *ʁˤaža birikːa* (*Heracleum apiifolium*). When she eventually picks it, she does it quickly, so that she can manage the cows at the same time. She peels off the skin, gives some of the peeled parts to me and eats some herself. “Tasty isn’t it?”, she comments. “You peel it like a banana. Earlier in the season it was a bit better. This one is a bit too big.” She kicks away the rest of the *ʁˤaža birikːa* (*Heracleum apiifolium*). We continue up the hill, and after around half an hour we arrive at our destination, where the cows are supposed to stay and from where they know how to come back by themselves in the evening. Then we start the descent. We arrive at the little stream, where Malaykat leaves her bag. She takes her knife and plunges into thickets of various heracleum species, some of which are over 2 m high. She makes me stay, explaining that burns from heracleum happen very often, but not to her, so she can go.

“There are not many edible parts left,” she says, holding a bunch (they are too old and therefore a bit bitter, as she explained later) of *biričːa* (*Chaerophyllum aureum*). Although only the leaf stalk is eaten, she carries the whole long plants (cut at the base) to the village. “It has a strong smell, doesn’t it?”, Malaykat says, smelling the pile.

Malaykat stops to show me *qːaˤnala čutni* (*Malva neglecta*). She grabs an unripe fruit in her fingers, peels it off the calyx and eats it. “We used to eat it a lot as kids, and I still like it. And we collect the leaves for *ħuˤlkni*/*chudu*,” she says as she tears two leaves off the plant.

“Oh and this.” She reaches up high and cuts a plant off of a rock. It is called *sirič’ič’*. Malaykat’s sister from Druzhba ate it a lot. “Is it good for something?”, I asked. “No. just for eating.” *Sirič’ič’ni* (*Sedum spurium*) is eaten on the spot. Malayakat did not collect it for her family. She just stopped after we set off and said to me, “Maybe just grab one for Oleg” (our co-researcher).

The day has already started in the village. Hadjilay was sitting at the *godekan,* the meeting point in the village, near the House of Culture and public bath, which has been falling apart since the 90s. Malayakt took *biričːa (Chaerophyllum aureum*) from the pile and gave one to him. He began to peel it.

Malaykat sat down and told him where we went and what we collected on the way. The *biričːa* (*Chaerophyllum aureum*) that was left over from the meeting at the *godekan* was taken home, but nobody ate it afterwards. The same situation happened a couple of times: when not eaten immediately after being brought home, snack-plants were never eaten afterwards.

Two days later, kids from the village collected some *ʁˤaža birikːa* (*Heracleum apiifolium*) near the village, and before they started eating it themselves, they gave each person at the *godekan* a share (see photo [Aditional file 4]). There were not enough plants for everyone, so they went on to collect more.

The respect for elders was expressed through the sharing of wild leafy-vegetables,. Not only were the plants shared at the *godekan,* but also the women who collected the plants usually showed what they had brought from the trip to respected elderly men (*aksakals*) in the village, who confirmed both the names and the uses of the plant. It was one of the few areas where typically female knowledge was publicly consulted with men, who usually just nodded.

On the return from one of the forest walks with Patimat, my bag full of plants became an object of a vibrant discussion at the *godekan*. Hadjilay (the eldest man in the village) took each plant from us, smelled it and confirmed the name and the use. Then the oldest women touched it and re-affirmed what had been said. In some cases the names were discussed, with some people claiming that the plants had other names. The knowledge about wild leafy vegetables seems quite individualized, with same names circulating in one family, but not necessarily in neighboring households. This does not necessarily mean that this knowledge is dying out. Our interlocutors had practical skills and collected the plants, but some of them did not name them. This may be a result of the way this knowledge is made and shared (cf. [37, 38]). The second reason for differentiation of names may be due to differences in languages spoken in the village. The Tselibki language might have influenced the Shiri plant names, especially in the linguistically mixed households. Yet, although different names were claimed, eventually a consensus was reached, and I was given the “one proper name” by Hadjilay or one of the elder women. I could not help thinking that sometimes the name was invented on the spot, just as if naming all plants used in the village was not as important to the locals as it was to us.

Though men’s knowledge of plants is significantly less extensive than women’s, in cases when women lack a word or knowledge, they most often refer to the men, as those who speak Russian better, are more educated in schools, etc. Here, as has been documented in many other places in the world, women are largely responsible for the vertical transmission of traditional botanical knowledge [39].

#### Local narratives

Local plant knowledge in Shiri is seen as a local form of social capital, something to be proud of. It has not always been so: in an attempt to reach the goal of modernization, the Soviet Union provided women with different kind of work outside the home. Very often this resulted in the lack of time for plant collection: −women had to both work and take care of household chores [40]. With the fall of the Soviet Union, various discourses about nature and naturalness appeared and became widespread [33, 41]. Programs on healthy lifestyles, traditional medicine, etc. were shown on TV, while journals like ZOZh (*Zdorovyi Obraz Zhyznii;* En. Healthy Way of Life)^3^ were full of “healthy” recipes and homemade remedies. The sources of knowledge and skills began to change in many areas of the former USSR [42]; compare [21] for other parts of Europe. However, this did not affect Shiri very strongly. Most locals’ knowledge about wild leafy vegetables comes from their older kin (oral, vertical transmission [43]). Nevertheless, more and more often new ideas arrive in Shiri from the lowlands where discourses on nature and naturalness take the form of nostalgia for the old homeland and plants that people do not have access to anymore.

*My sister-in-law misses ʁˤaža birikːa [Heracleum apiifolium] most, as it does not grow in the lowlands and you cannot transport it. We only send them greens for ħuˤlkni (woman, 42).*

A link may be made between nostalgia for plants^4^ and food nostalgia (see for example Melissa Caldwell on food nostalgia in Russia [44]). Shiri people are proud of the fact that they live in the village where there are so many plants that they see as exceptional, and where everything is “clean and fresh.” Part of the local identity is being formed through such discourses (cf. [33, 34]).

Migrants who learn about new plants from TV and magazines want to find them in Shiri. So they often ask kin to find the plants for them, because local knowledge and knowledge of the location of specific plants is very much valued and appreciated, by the migrants. –“Book/TV” knowledge brought up from the lowlands merely adds to what the locals already know cf. [21].

Though the inhabitants of Shiri acknowledge the hardships of life in winter, they eagerly grasp upon the discourses of trying to justify their choice of “staying behind” in the village with “ecology” (for more on the local understanding of the word in the post-Soviet context, see for example [33, 45]).

*Everything is ecological here, our plants, our air. When I go to the lowlands I suffocate and I can’t eat anything because I throw up (woman, 42).*

Ecology is listed as one of the first causes for not migrating despite the various opportunities. Shiri people are “historically” better off than the Dargi in neighboring villages (there are various reasons for the Shiri’s relative wealthiness, but that is beyond the scope of this article), therefore migration to the lowlands is available to most of them (some older inhabitants migrate to the lowlands for the winter, while they spend the summer in Shiri), yet most of the people decide not to leave the village, and plant collection is among the practices listed as one of the reasons.

*If we leave who is going to collect greens for them? You can’t find them in the lowlands. They have nothing there.*

At the same time, they acknowledge that their kin in the lowlands have better, more diverse gardens with tasty varieties of vegetables and know how to take care of them. In Shiri some households cultivate home gardens, but most of them are not taken care of and Colorado potato beetle (*L. decemlineata*) eats most of the crops. Some of these plots were established in the period of Perestroika, when people began to cultivate land in response to the economic crisis, even in the cities. In many parts of the former USSR, the land was given away, especially for use as dachas, in order to increase food security and to keep people busy; see for example [33, 46]. At the time of the research, only one women in Shiri was proud of her “ecological” garden, while others mentioned it only when asked, and with a look of embarrassment. Once cultivated, most of them were partly or totally neglected. Most women saw no reason to put effort into growing vegetables that were usually small and poor anyway.

The discourse of naturalness is strictly linked to the health discourse (for the post-Soviet context of these discourses, see for example [45]). Locals also appreciate wild leafy vegetables for their nutritional value and vitamin content. They see them as healthy. Obviously, the division between medicinal and edible plants is rarely clear and strict (see for e.g., [16, 47]).

*They say all the plants we eat here are good for your health: this is why people get more infections in the lowlands. Sːisːupi [Allium victorialis] has the most vitamins (woman, 78).*

They also acknowledge the importance of wild leafy vegetables in early spring when fresh vegetables are not widely available (or are expensive). Among the greens they felt the need to eat after winter, the most often listed were: the raw root and stems of *haq’ul* (*Arctium lappa*), the bulbs of *podsniezhnik* (*Galanthus* spp.), *ħuˤlkni* with *ʡaˤʁˤamura* (*Cerastium davuricum*) and *guržinakːˤi* (*Oberna multifida*).

Infrastructural problems are also an issue here (for older or car-sick villagers, the bumpy road between Shiri and Kubachi was the real obstacle preventing them from visiting the bazaar too often), though sometimes they were listed more as a justification for a certain traditionally inherited behavior (like eating more greens in spring than in late summer) than a real problem, because kin or neighbors were always ready to supply elders with the goods they needed.

Older inhabitants of Shiri were not sure about using some vegetables because they rarely appear in their everyday diet (some appear in holiday dishes like *moskovskiy salat*^5^), while wild leafy vegetables are at hand, available when one feels like eating *ħuˤlkni* with greens.

*Our ancestors ate those because they had no vegetables and even now our women prefer ħuˤlkni with greens […] Vegetables in Kubachi [neighboring settlement with shops and bazaar] are not always fresh and you have to make a trip there (man, 81).*

A closer look at the narratives revealed that most often the nutritional value of leafy greens and their perceived exceptionality were emphasized, which makes them an important constituent of local identity in times of increased migration. The analysis of practices revealed that wild leafy vegetables tend to be important in both caring for co-villagers and migrants and expressing the respect for elders.

## Conclusions

Knowledge of wild leafy vegetables is common in the village of Shiri. There are 24 folk species of wild leafy vegetables gathered there. All of them are in everyday use by most members of this small community. As many as 72 % of all greens taxa used are consumed as snacks. The number of snack-plants seems to be very high in the European context [24]. Around 36 % of leafy-vegetable taxa recorded are used for cooking (mainly *chudu* and *kurze*). Some plant taxa are used in more then one way. Knowledge of cooked greens is more evenly spread than knowledge of snack-plants, particularly among females living in the village.

In many places in Europe traditionally characterized by elaborate knowledge of green vegetables, this knowledge is diminishing (e.g., [24, 48]). In our research site, wild leafy vegetables have high significance but migration to the lowlands will definitely influence it. “Healthy” and “ecological” lifestyle trends may have their impact as well.

Wild leafy vegetables are a significant element of everyday social life in Shiri in regard to mutual care, respect for elders and local identity and pride.

Knowledge about plants is mostly the women’s domain cf. [5]; it is both individual and strongly embodied, yet there is also a specific social dimension to it which goes beyond casual skills and the exchange of knowledge among those practicing plant knowledge. Though they do not collect and prepare wild greens, elderly men are customarily asked about plants collected and brought to the village. They serve as consultants, and they may suggest the name of a plant, even if they have to invent it on the spot, yet women are the ones who practice skills and local knowledge about plants. The names of the plants are publicly discussed with elders and are not always fixed: sometimes they are even invented on the spot both by men and women. Much research also shows that men often collect plants from ‘men’s spaces’ and women collect from ‘women’s spaces’ [12]. The spaces farther from the village (in surrounding mountains and valleys) are shared by both genders. As both men and women were involved in everyday cattle herding, they had an opportunity for plant collection outside the village. Snack-plant collection is performed by both genders in similar places, so there was no gendered space division in this context. In Shiri, we identified a place near the houses where greens for *chudu* grow as a women’s space. This is the space men knew little about. Generally women's knowledge about plants was more extensive then men’s.

In Shiri care is often expressed by sending wild leafy vegetables to kin in the lowlands. This helps to sustain social ties between migrants and Shiri inhabitants. Wild leafy vegetables help express love and care to one’s family, kin or strangers, who eagerly receive packages that they can later share with those around them.

Wild leafy vegetables are also spoken about quite frequently. There is a strong discourse of health, naturalness and “ecology”, that is adding value to local knowledge about wild leafy vegetables. Living in a “natural” environment full of appreciated and loved local wild leafy vegetables and being able to provide them for others is very important. It also constitutes an important element of the local identity. In times of migration to the lowlands, Shiri people also fear that one day, when the old people die, the young ones will leave the village looking for a better life. They perceive themselves as the last ones struggling to live in difficult climatic and environmental conditions, yet proud to be there, to stay where their ancestors lived and died, where they cared for their families, and where they collected wild leafy vegetables and other plants. When the older generation of women passes away, so will the knowledge about wild leafy vegetables and medicinal plants. Their knowledge is only partly passed on to the younger generation, which is no longer interested in long, often exhaustive walks, and prefers to have a pack of chips for a snack. Bussmann and colleagues made similar observations in Georgia: “young villagers know only plants that grow in the village. The plant knowledge is concentrated in women, and men rely on that knowledge when needed [5]”.

We find it crucial to continue our research and document both the specimens and social practices connected with their collection, both in Shiri and in other scarcely populated neighboring villages in the Dakhadaevsky region. Daghestan is the last place in the whole Caucasus where mountain slopes are populated throughout the whole republic. While it is likely to change, more research is needed before most of the mountaineers moves to the lowlands.

## Additional files

Additional file 1:
**Map of Daghestan.** Map of Daghestan prepared for the project “Documenting Dargi Languages in Daghestan: Shiri and Sanzhi”. (JPEG 493 kb) Additional file 2:
**View on Shiri village.** The photo was taken in June 2014 in the center of Shiri, Dakhadaevsky region, Daghestan, Russian Federation. Author: Iwona Kaliszewska. (JPEG 461 kb) Additional file 3:
**Preparation of a pie with greens in Shiri village in Daghestan.** The video was recorded on July 7th 2013 in Shiri, Dakhadaevsky region of Daghestan, Russian Federation. Patimat (woman, 81) was preparing a pie out of greens for her family. She collected wild leafy vegetables for *chudu* earlier this day. *Chudu with greens* is an everyday dish prepared predominantly in spring and rarely on special occasions like weddings when dishes with meat are more valued. Author: Iwona Kaliszewska. (mp4 15284 kb) Additional file 4:
**Sharing wild greens in the center of Shiri village.** The photo was taken in June 2014 in the center of Shiri. Young boys collected various Apiaceae species in the village. Before eating it themselves, they shared it with other villagers who sat at the *godekan,* the central meeting point in the village. Author: Iwona Kaliszewska. (JPEG 708 kb) Additional file 5:
**Forest walk for** ***sːisːupi*** **(** ***Allium victorialis*** **).** The photo was taken in the area surrounding the villageo of Shiri when Patimat (women, 81) went on a day-long walk to collect *sːisːupi* (*Allium victorialis*). She collected it both for cooking for her family and to send it in dried form to her kin in the lowlands. Author: Iwona Kaliszewska. (JPEG 741 kb).

## Footnotes

1 Documentation, grammar and dictionary of Shiri languages is one of the goals of the project: “Documenting Dargi Languages in Daghestan: Shiri and Sanzhi” financed by the Volkswagen Foundation. This is work in progress. Some results can be viewed on the project’s website: http://www.dargwa.kaukaz.net [10].

2 It is difficult to find reliable source to confirm the toxicity of *Crocus reticulatus* and *Fritillaria collina*, but they belong to the genera represented by many toxic species [29].

3 Plants presented in this magazine come from all over the former USSR, but mainly Russia, and thus represent high habitat diversity. Moreover, illustrations in the magazine are very scarce, usually black and white, and are not really helpful in plant determination. (More on the subject [30]).

4 Perhaps the term ‘plant-nostalgia’ would be appropriate here, yet this would require a separate discussion which exceeds the scope of this article.

5 Also known as Olivier Salat: http://www.sras.org/Russian_olivier_salad. Recipe: http://www.enjoyyourcooking.com/salads/russian-salad-olivier.html
