# Supplementary figures and images for: The social context of wild leafy vegetables uses in Shiri, Daghestan
Source: J Ethnobiol Ethnomed. 2015 Aug 11;11:63. doi: 10.1186/s13002-015-0047-x (PMC4542102; doi:10.1186/s13002-015-0047-x)

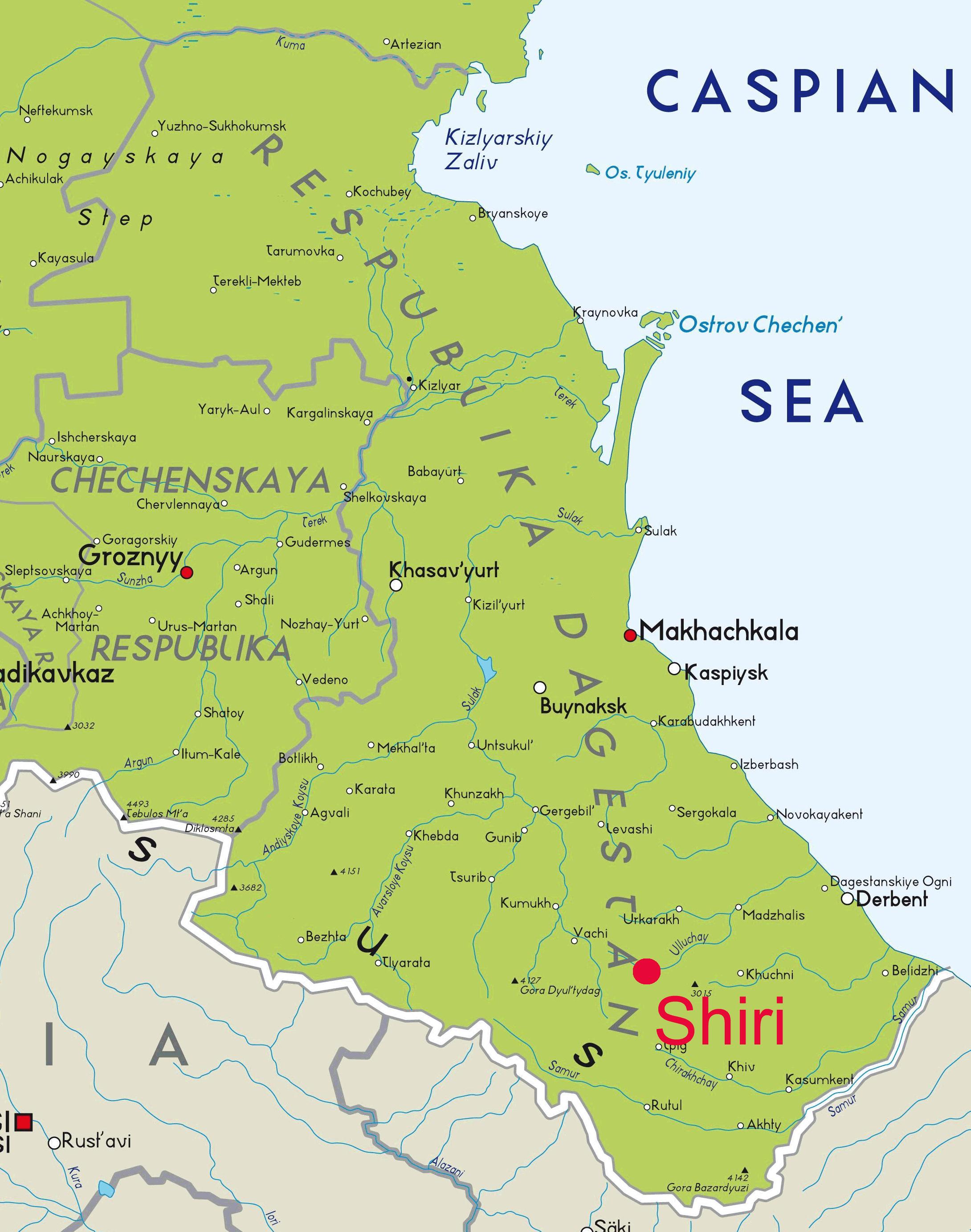

Supplement: Additional file 1: — Map of Daghestan. Map of Daghestan prepared for the project “Documenting Dargi Languages in Daghestan: Shiri and Sanzhi”. (JPEG 493 kb) [file 13002_2015_47_MOESM1_ESM.jpeg]

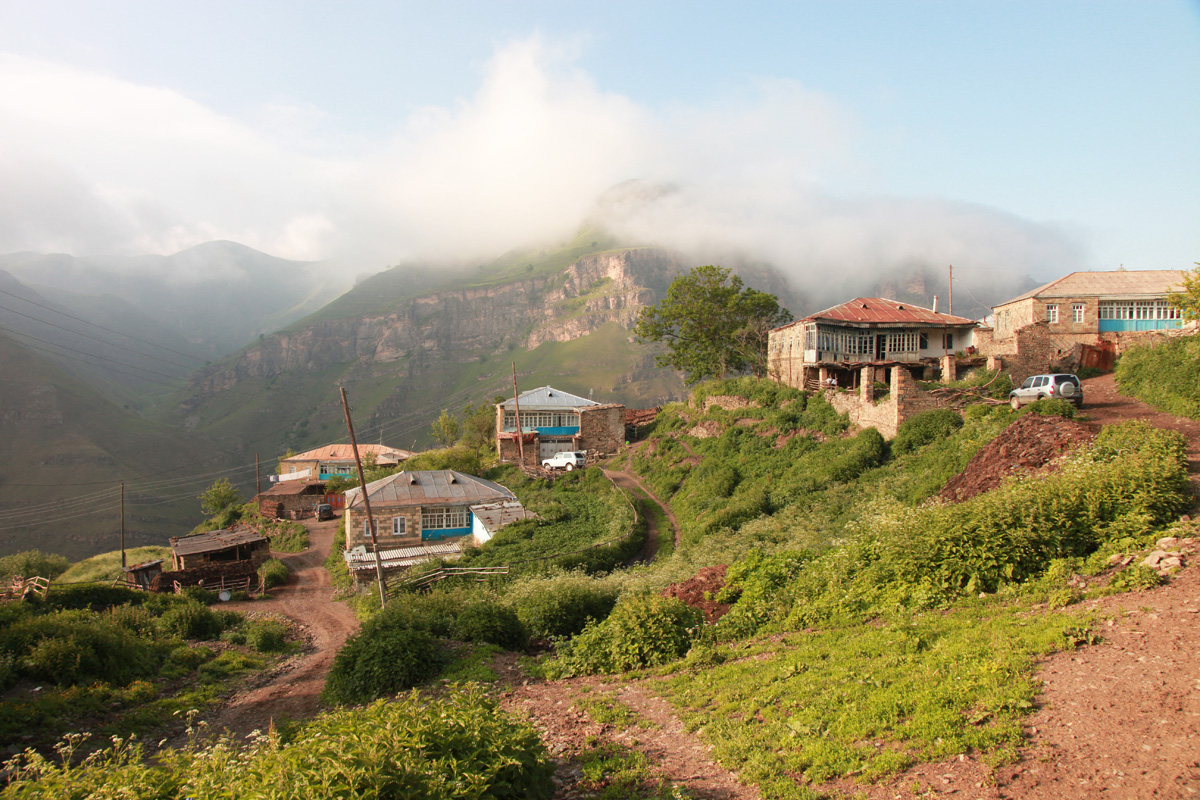

Supplement: Additional file 2: — View on Shiri village. The photo was taken in June 2014 in the center of Shiri, Dakhadaevsky region, Daghestan, Russian Federation. Author: Iwona Kaliszewska. (JPEG 461 kb) [file 13002_2015_47_MOESM2_ESM.jpeg]

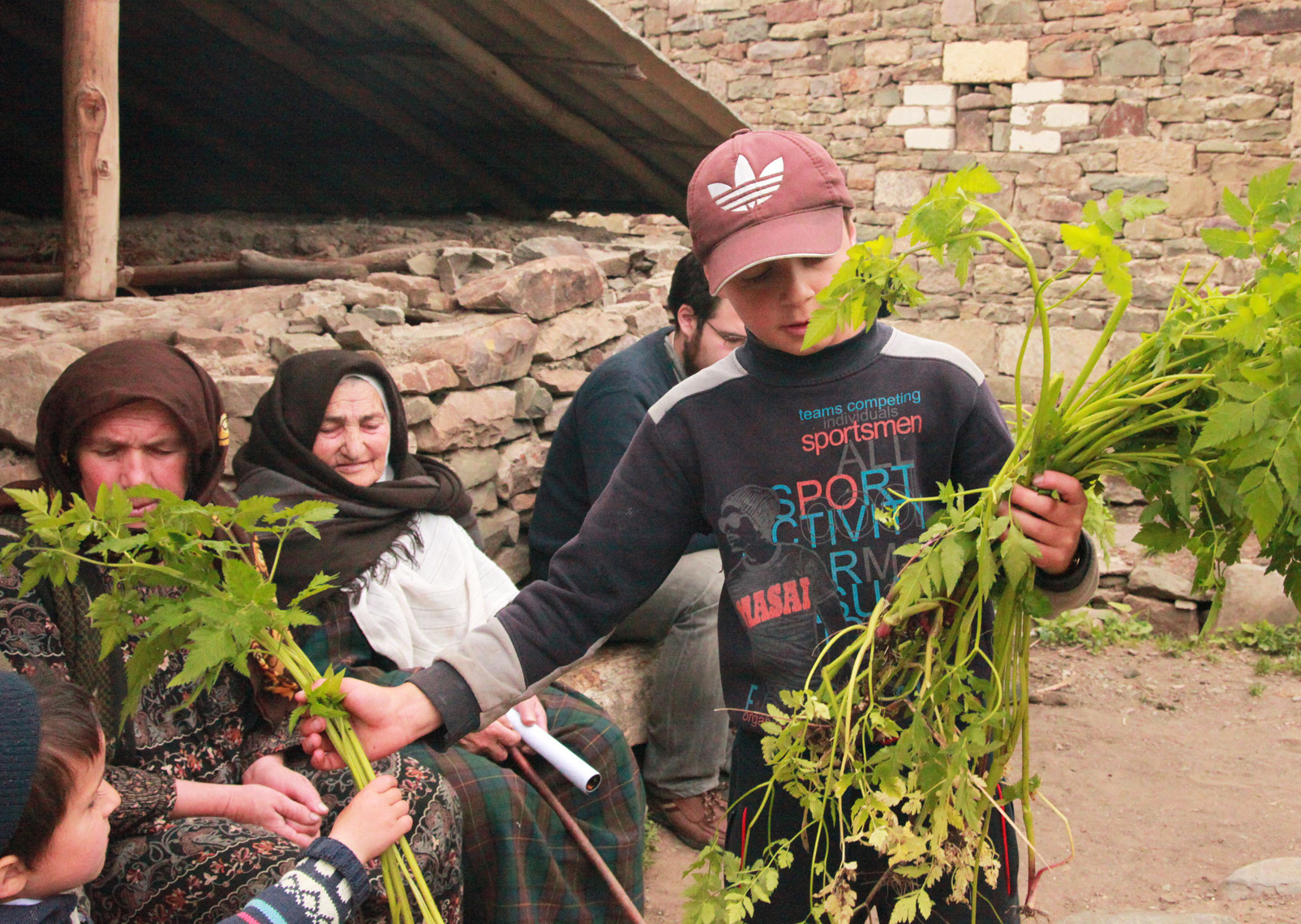

Supplement: Additional file 4: — Sharing wild greens in the center of Shiri village. The photo was taken in June 2014 in the center of Shiri. Young boys collected various Apiaceae species in the village. Before eating it themselves, they shared it with other villagers who sat at the godekan, the central meeting point in the village. Author: Iwona Kaliszewska. (JPEG 708 kb) [file 13002_2015_47_MOESM4_ESM.jpeg]

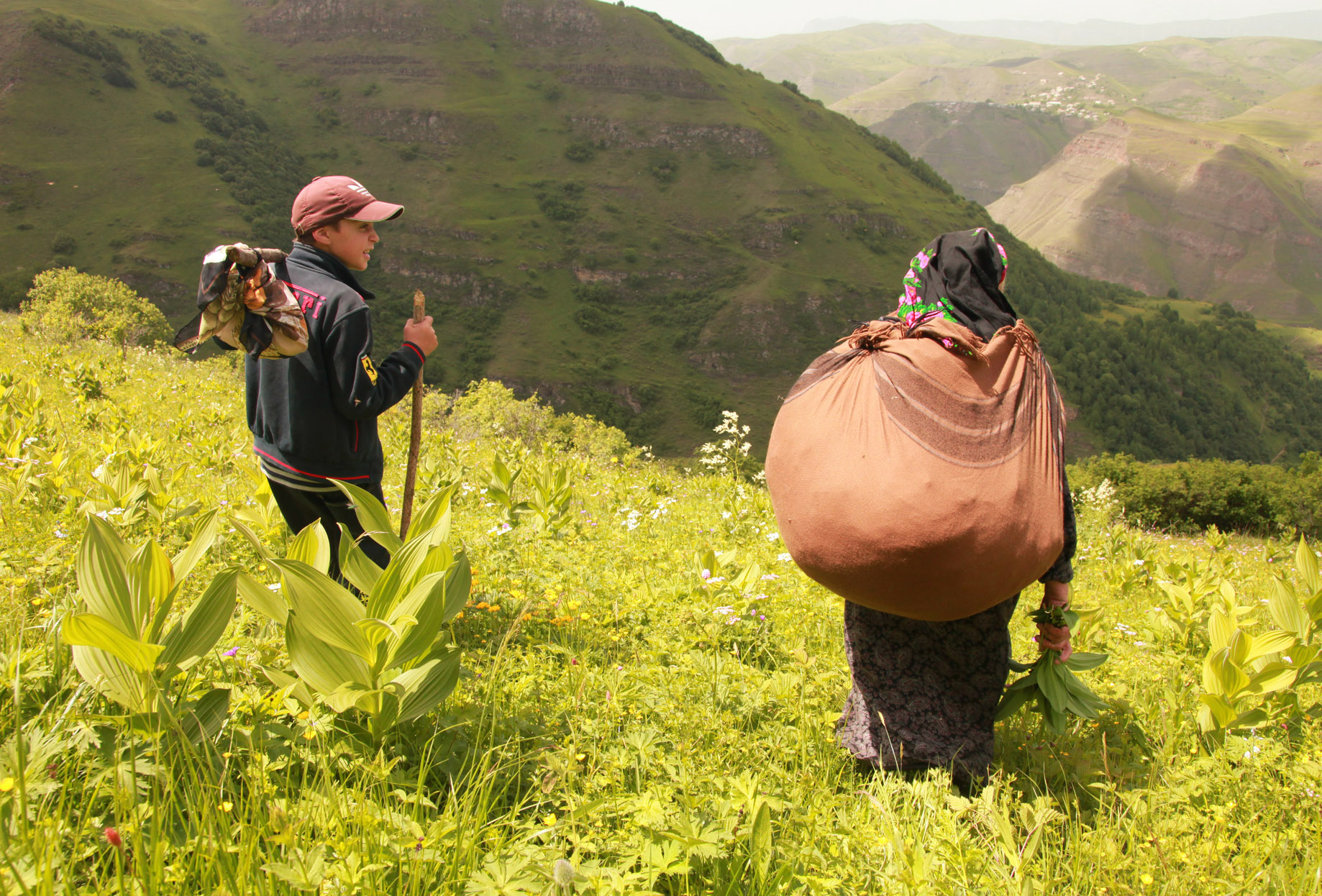

Supplement: Additional file 5: — Forest walk for sːisːupi ( Allium victorialis ). The photo was taken in the area surrounding the villageo of Shiri when Patimat (women, 81) went on a day-long walk to collect sːisːupi (Allium victorialis). She collected it both for cooking for her family and to send it in dried form to her kin in the lowlands. Author: Iwona Kaliszewska. (JPEG 741 kb). [file 13002_2015_47_MOESM5_ESM.jpeg]
